# Combating Barriers to the Development of a Patient-Oriented Frailty Website

**DOI:** 10.2196/53098

**Published:** 2024-05-28

**Authors:** Brian Greeley, Sally Seohyeon Chung, Lorraine Graves, Xiaowei Song

**Affiliations:** 1 Fraser Health Authority, Surrey, BC, Canada

**Keywords:** frailty, frailty website, patient-oriented assessment, community-dwelling older adults, internet security, privacy, barrier, barriers, development, implementation, patient-oriented, internet, virtual health resource, community dwelling, older adult, older adults, health care professional, caregiver, caregivers, technology, real-time, monitoring, aging, ageing

## Abstract

This viewpoint article, which represents the opinions of the authors, discusses the barriers to developing a patient-oriented frailty website and potential solutions. A patient-oriented frailty website is a health resource where community-dwelling older adults can navigate to and answer a series of health-related questions to receive a frailty score and health summary. This information could then be shared with health care professionals to help with the understanding of health status prior to acute illness, as well as to screen and identify older adult individuals for frailty. Our viewpoints were drawn from 2 discussion sessions that included caregivers and care providers, as well as community-dwelling older adults. We found that barriers to a patient-oriented frailty website include, but are not limited to, its inherent restrictiveness to frail persons, concerns over data privacy, time commitment worries, and the need for health and lifestyle resources in addition to an assessment summary. For each barrier, we discuss potential solutions and caveats to those solutions, including assistance from caregivers, hosting the website on a trusted source, reducing the number of health questions that need to be answered, and providing resources tailored to each users’ responses, respectively. In addition to screening and identifying frail older adults, a patient-oriented frailty website will help promote healthy aging in nonfrail adults, encourage aging in place, support real-time monitoring, and enable personalized and preventative care.

## Introduction

Technological advances, principally computer-aided assessments and electronic health information exchanges, have enormous promise to promote healthy aging. Fortunately, older adults (ie, those aged ≥65 years) are open to using new technologies [1]. Thus, health care is adopting technologies that support real-time monitoring to promote personalized and preventative care [2].

Frailty is an age-related dynamic state characterized by deficits across multiple physiologic systems with increased vulnerability to stressors [3,4]. Consensus guidelines state that early detection of frailty is key to its management [5,6]. To this end, technology-based frailty tools have allowed health care professionals to screen, assess, identify, and develop care plans for frail persons [7-11]. Yet, physician-oriented frailty screening tools are resource intensive.

A patient-oriented frailty website is the next step in frailty care [12]. In Canada, approximately 23% of community-dwelling older adults are frail [13]. Although primary care frailty screening tools used by health care providers show promise in the identification and management of frailty [14], many community-dwelling older adults do not have the ability to undergo in-person frailty screening. Therefore, patient-oriented frailty screening tools in the form of websites and apps should be developed and implemented.

The concept of a patient-oriented frailty website is straightforward. Individuals go to a secure frailty website where they answer a series of health-related questions. The website, in turn, generates a frailty/health summary and score based on the user’s responses. In addition to a score, the website could also inform the user of their frailty status and whether they should seek medical counsel. Upon consent, users can share their responses and score with their care provider. In this capacity, a patient-oriented frailty website could identify those that are frail or at an increased risk of becoming frail. The website would be inclusive and encourage *all* older adults, regardless of health status, to participate. For healthy older adults that show no signs of frailty, the website would promote and reinforce their current healthy lifestyle and serve as a baseline. In this way, a frailty website is similar to the standard practice of well-baby visits—periodic examinations that screen for potential developmental health complications [15]. Therefore, well-baby visits and web-based frailty assessments are tools for the prevention and treatment of health issues that occur early and late in life, respectively.

To understand the interest in a patient-oriented frailty website, we conducted a planning project consisting of 2 components. In May 2023, we held a web-based educational symposium on frailty [16]. Experts in frailty spoke on the importance of frailty assessment and identification, approaches to preventing and mitigating frailty, frailty in primary care, and factors common to those who age gracefully beyond the age of 85 years. Recruitment for symposium audience and discussants was done via posters, which were distributed and published in local health authority newsletters, at academic institutions, and at a volunteer recruitment site for health research using REACH BC (the *Ethical Considerations* section provides more details). The audience was Canadian clinicians, researchers, caregivers, and community-dwelling older adults. Before and at the symposium, we invited interested audience members to participate in a discussion session scheduled 2 weeks later; individuals were excluded if they had not seen the symposium. Interested care providers, caregivers, and community-dwelling older adults returned to 1 of 2 discussion sessions. One discussion session included 7 caregivers and care providers, whereas the other session included 6 community-dwelling older adults; sample sizes were determined by best practices [17-19]. In contrast to a traditional qualitative research where findings are summarized then supplemented with participants’ quotes, in this viewpoint paper, we summarize the main findings of the discussion groups, highlighting the major barriers to developing a patient-oriented frailty website. To benefit other researchers developing similar health websites, we also provide potential solutions as observed throughout the literature and discuss caveats to those solutions, as summarized in Table 1.

**Table 1.** Barriers, their potential solutions, and caveats to those solutions regarding the implementation of a patient-oriented frailty website.

Barriers and solutions		Caveats
**Completing a frailty assessment on the web is restrictive to frail persons.**		
	Caregiver assists or completes assessment on frail person’s behalf.	Not all frail older adults will have a caregiver.
	Distribute low-tech (ie, paper) frailty assessments.	Requires a set of complex steps a frail person cannot do.
**There are privacy and security concerns.**		
	The website needs to be hosted from a reputable source with clear intent on its use.	It is unknown how users will perceive the trustworthiness of a frailty website.
**Users may restrict researchers from health data analyses.**		
	Implement federated learning.	Federated learning is still relatively novel and may be susceptible to attacks.
**Users want to complete the assessment quickly.**		
	Reduce the number of frailty questions.	Fewer questions may compromise the clinical usefulness of the assessment.
**Health care providers need to know how to interpret and apply the information to care for frailty.**		
	Develop models to treat frailty; implement training for health professionals.	This solution requires significant resources.
**Being labeled as frail is counterproductive to combating frailty.**		
	Educate users on frailty and reframe frailty in a more positive light (eg, well-being).	It is unknown how users will respond to their (potentially negative) scores.
**Users want health resources tailored to them in addition to a frailty/health assessment.**		
	Provide additional resources tailored to users’ responses.	Users may be deterred from seeking the expertise of medical professionals.

## Ethical Considerations

This planning project received an exemption from the Fraser Health Research Ethics Board as it fell under quality improvement and evaluation studies. Stakeholders provided informed consent prior to the discussion session for review, following the Fraser Health guidelines. All stakeholders had the ability to opt out at any time for any reason and were reminded of this right prior to the start of the discussion session. Both discussion sessions were recorded for the purpose of transcription offline. Videos of the 2 discussions are stored on a secured drive that is both password protected and can only be accessed by approved Fraser Health employees. Stakeholders were compensated CAD $25 (US $18.27) for engaging in the symposium and 1 discussion session.

## A Website That Assesses Frailty Is Restrictive to the Frail

The most substantial issue involving the implementation of a patient-oriented frailty website is its restrictiveness. A website targeting a subpopulation assumes that the targeted audience can access the website. On average, internet use among Canadian older adults is 68%, but it is only 41% for those aged ≥80 years [20]. In addition to age, health and frailty status are also related to internet and computer use. For example, 73% of Canadian older adults in excellent or very good health use the internet, whereas 62% of older adults in fair or poor health use the internet [20]. Likewise, another group found that the frailer a person was, the less likely they were to use a computer [21]. Thus, attempting to reach frail older adults through a website is a major hurdle.

A seemingly simple solution to this barrier is targeting caregivers (eg, partners, family members, friends, colleagues, or neighbors) and having caregivers assist or complete the questions on the frail person’s behalf. However, this solution only works for those with caregiver support. In one study based in the Netherlands, having a primary caregiver was reported in 32% of older adults who visited the emergency department [22]. This figure may be slightly lower in Canada. It is estimated between 23% [23] and 28% [24] of older Canadian adults have caregivers. Consequently, there is a chance that most frail community-dwelling older adults would not have assistance using a computer, navigating to the frailty website, and answering a series of health-related questions. Clearly, increasing caregiver support is one approach to increasing internet access among frail older adults.

Another solution to combat the restrictiveness of a frailty website is to also provide a paper version to frail older adults. Despite acknowledging the importance and benefits of digital screening tools, older adults have suggested that low-tech alternatives should also be available [25]. It would be convenient to make a paper pamphlet that has the same information and questions as a frailty website. This way, both paper and web-based frailty assessments would be interchangeable, allowing for conversion from one medium (ie, paper) to the other (ie, website) and vice versa. Pamphlets can be widely distributed and made available at family physicians’ and nurse practitioners’ offices, wellness and health clinics, pharmacies, and even mailed directly to older adults (eg, using Canada Post’s Precision Targeter). However, there are concerns regarding this approach as well. Assuming frail community-dwelling older adults obtained access to the frailty pamphlet survey, they would still need to fill it out (accurately), return it, schedule an appointment, and visit a health care professional. Some of these barriers can be mitigated by including a return address with free postage, using free door-to-door shared ride services, or scheduling a telehealth appointment. However, this process requires a series of complicated steps and older adults with mild cognitive impairment will be unable to complete them [26]. Other means of reaching frail persons are needed, but without significant assistance, it is unlikely that these individuals will be screened for proper frailty care.

## Older Adults Have Internet Privacy Concerns

Despite a positive attitude toward web-based health services [27], older adults are concerned about privacy, especially when it is unclear by whom or how their medical data will be used [28]. For example, the probability of being identified was the single most important attribute when older adults considered internet privacy, even though sharing their medical data was viewed positively if it was to be used for science and the development of novel care and treatment [29]. The hesitancy of older adults to share their medical data reveals the need for clearly communicating how their information will be used—if they are to be comfortable consenting to its wider use. Similarly, a previous study found that older adults were more willing to share their data (eg, demographics, family relations, economic status, physical and mental illnesses, family history, medication, and health care service use) with family and hospitals compared to researchers or government agencies [30]. Public trust in the government is complex. For example, despite only 19% of Americans trusting the federal government, 70% viewed the US Food and Drug Administration and US Centers for Disease Control and Prevention favorably [31]. Thus, hosting a patient-oriented frailty website on a health authority or a particular branch of government, as opposed to a commercial site, with the clear intent to promote health, may increase the likelihood of the widespread adoption of a web-based frailty assessment tool among older adults. However, more work is needed to fully understand the actual adoption (as opposed to the hypothetical adoption) of a frailty website among older adults and how to securely transfer a user’s medical responses to a health authority without compromising privacy if the user consents.

## Users’ Restriction of Their Data May Limit Health Data Analyses

The reluctance for individuals to share their health data for research purposes is understandable and may dictate the use of a frailty website. Researchers and health authorities may bemoan the decision for users to protect their data, which, in turn, may restrict our understanding of frailty. For example, knowing the frailty status and location of older adults who completed a web-based frailty assessment could reveal concentrated areas of frail older adults, suggesting environmental or societal risk factors and the need for additional services to be deployed to those locations. Realistically, this type of analysis can only be done with a user’s location and health data. Still, it is important to remember that the personal health data acquired from any website belongs to the user. It is at the discretion of the user (assuming they have the cognitive faculties to consent in the first place, a topic not discussed in our sessions) whether to share their data.

Hence the success of a frailty website is contingent upon the implementation of a privacy-first approach. A privacy-first approach underscores the users’ ownership of their data, using dynamic identifiers and storing data locally (ie, on a smartphone or computer), as opposed to a data-first approach, which prioritizes the retention and distribution of data, typically in a centralized location [32]. However, a happy medium exists where users can keep and protect their data while researchers and health authorities can advance frailty care through modern analysis.

New advances in data analysis [33] have been developed and used to underscore privacy-first approaches. Federated learning, for example, is a machine learning model that aggregates training results from multiple sources to create a consensus model without the need for data to leave a given device or system. A recent study found federated models achieved the same accuracy, precision, and generalizability as standard centralized statistical models using a variety of health data [34]. As a specific example, federated learning was used to predict treatment response in breast cancer patients using data behind a hospital’s firewall [35]. Emerging technologies and analyses have made it possible to have a patient-oriented frailty website that both ensures the privacy of the user and allows for analyses that will usher in better frailty care. A caveat to this is that federated learning is relatively new and will take time to implement across health authorities, and it may be prone to specific types of attacks [33].

## Users Want to Complete a Frailty Assessment Quickly

A frailty website needs to be efficient and user-friendly [12] while adequately collecting health information that can assess frailty [3,4]. After the user completes a series of health-related questions on a frailty website, the website should produce a score (eg, “Your frailty score is 42/100, consider making an appointment with your doctor” or “Your biological age is 71, 6 years older than your actual age of 65”). One scoring approach could be a multisystem deficit-accumulation frailty index [36,37], which subscribes to the idea of an accumulation of deficits and is scored between 0 and 1, with 0 being no deficits present and 1 being all deficits present and fully expressed (in reliaty, the score seldom exceeds 0.7, a limit of deficit acculation); this approach has acceptable validity, reliability, and diagnostic test accuracy [38]. One benefit of a frailty score derived from a frailty index is that it can be interpreted by nonexperts. However, because the accumulation deficit model subscribes to the idea that frailty is a multisystem state, ideally a frailty website that adopts this approach would require 30 to 40 questions [39] across multiple domains (ie, physical, cognitive, psychological, and social). Research has shown that people are more likely to complete a survey if it takes 15 minutes or less [40]. In the context of a frailty website, completing 30 to 40 questions in 15 minutes would require users to spend 23 to 30 seconds on each question. Therefore, inaccurate responses and the respondent feeling rushed, frustrated, and stressed can be a concern.

In an attempt to make the website user-friendly, the number of questions would have to be reduced, potentially compromising its clinical utility [41]. A frailty index comprising fewer than 30 variables and questions can still be useful. In one study, researchers found that a frailty index constructed using 23 variables was just as accurate as one constructed using 70 variables [42]. An alternative approach to written multiple-choice questions may be a pictorial frailty assessment. The Pictorial Fit-Frail Scale [43] was recently developed for this purpose. It is fast (it took patients 6 minutes to complete) and comprehensive (the assessment covered 14 domains). However, agreement rates among Canadian and UK health care professionals were low (32% agreement for social, 44% agreement for mood, and 59% agreement for function), and it is unclear how patients understood each domain (averages were taken across 146 patients, caregivers, health care professionals, and general public participants) [43]. In contrast to the frailty index with precise grading of frailty, the phenotype model of frailty includes only 5 variables. Yet, the phenotype model requires grip strength and walking speed, measures that cannot be easily tested and may not be safe for many older adults to complete in their own homes. A methodological consensus regarding the definition of a frailty index, the variables that comprise it, and how they are scored has been encouraged [44-46].

## Frailty Score Needs to Be Interpretable

In addition to a methodological consensus, output from a patient-oriented frailty website must be sufficient and interpretable for all health care professionals irrespective of location. For example, health care professionals across Canada would need to know what a frailty score of “42/100” means and how to prescribe the appropriate care. Our modern health care system is well designed for treating diseases but not for embracing the unique challenges of frailty among older adults, a population that experiences complex health issues [47].

Fortunately, the development of novel models [48] and guidelines [49] that address frailty in primary care is already underway. Moreover, frailty training would be required for physicians to prescribe appropriate frailty care. A systematic review found that there were limited frailty training programs for health professionals; however, the programs that did exist effectively increased frailty knowledge and competence in frailty assessments [50]. Although this would require significant resources, postponing or reducing frailty would result in a significant reduction in health care costs [51] and would pay for itself.

## Being Labeled Frail Is Counterproductive to Combating Frailty

Numerous studies have agreed that there is a stigma around the concept of frailty and being labeled as frail. In one study, community-dwelling older adults reported that frailty was perceived as “approaching the end of their lives, malnourished and highly dependent on care” [52]. This same study also found that older adults are likely to reject the concept of frailty even when an objective measure may define them as frail [52]. Hence, a frailty website that labels a user frail may be counterproductive to the purpose of preventing and managing frailty.

In addition to education, another solution to combat the negative perceptions of frailty is to reframe the concept of frailty in a more positive light, using terminology such as “healthy aging” or “well-being” [52,53] or, within the context of a fit-frail score, “well-being score.” It should be noted, however, that positive output from a web-based frailty assessment (eg, a low fit-frail score, or a low biological age relative to chronological age) could also be counterproductive and have the same consequences as a negative output, deterring older adults from seeking medical care. Nevertheless, at least 1 study suggests otherwise. Among older adults who avoided medical care, 36% did so out of fear of a serious illness, and the likelihood of avoidance was greater in those who self-reported a poorer health status [54]. Ultimately, it is unknown how users will respond to negative or positive outputs from a frailty website, and more research is needed to understand the potentially complicated reactions older adults will have after receiving their personalized health score and summary. Regardless, it is of the utmost importance that older adults are comfortable, feel safe, and are motivated (not discouraged) when using a frailty website.

## Frailty Websites Should Provide Additional Health Resources

A frailty website should offer resources in addition to a health score and summary. A patient-oriented frailty website that gathers health information can be of great use for health care providers. It should also produce personalized resources based on the user’s responses [55,56]. For example, if a community-dwelling older adult is deemed to be at risk of becoming frail and their deficits are primarily in a physical domain (ie, they are slightly overweight, sleep less than 6 hours a night, drink >6 alcoholic drinks a week, and have hypertension but are cognitively normal), the website ought to provide links to resources to meet those care needs. In this scenario, the website could suggest resources such as how to start and maintain an exercise program and provide best sleep hygiene practices in addition to recommending that the user make an appointment with their physician to manage hypertension.

Self-management is a potentially viable, low-cost approach to addressing frailty, but this is a relatively unexplored topic in frailty care [53]. Additionally, caution must be exercised with self-management. For instance, individuals that are provided resources may be lulled into a false sense of security and feel that their health can be managed without regular check-ups by the expertise of trained medical professionals. On the other hand, older adults may be unmotivated to complete a series of health-related questions if a website does not provide any immediate, clear feedback other than a score. There needs to be a happy medium where older adults are motivated to use a frailty website but also supplement the website with a medical expert’s opinion. Future studies need to understand where that happy medium exists and incorporate it into frailty website development.

## Limitations

This viewpoint paper is not without limitations. One such limitation is the lack of quotes from discussants. While quotes are typically included in qualitative research to strengthen findings, our viewpoint explicitly draws attention to barriers and potential solutions to the development of a patient-oriented frailty website. Further, traditional focus group studies that include theme discovery supplemented with quotes will be needed to better understand and address these barriers. Another limitation is the omission of technological barriers, which have been published elsewhere [12,57]. One important technological barrier is search engine optimization—a process of making modifications to a website to increase its visibility. Well-tuned site optimization strategies increase traffic to health websites, whereas poor search engine optimization can affect older adults’ experience in frailty assessment and health interpretation simply due to a missing hyperlink to the website. Finally, the barriers reported here may not generalize to populations outside of Canada. However, some of the barriers reported here also exist for older adults in Switzerland [26] and the United Kingdom [53,56]. Future studies should attempt to recruit samples representing broader geographical regions to promote equity in global health care.

## Conclusion

A frailty website that can be used by the community to screen and identify older adults at an increased risk of frailty and health decline is an important step in geriatric care and public health. However, several barriers must be addressed in future research before the development of such websites. While some barriers have potential solutions, they come at a cost (eg, resources required for optimizing frailty models in primary care and patient-oriented frailty assessment training and support). Other potential solutions (eg, caregiver assistance and an accompanying paper-based frailty assessment) have their own challenges. Regardless, addressing these barriers, even partially, is a worthy goal. The early detection and management of frailty can lead to significant inroads to integrated care, benefiting the quality of life of older patients and their caregivers and the health of the aging population.
